# Handheld Raman Spectroscopy in the First UK Home Office Licensed Pharmacist-Led Community Drug Checking Service

**DOI:** 10.3390/ijerph20064793

**Published:** 2023-03-08

**Authors:** Anthony Mullin, Mark Scott, Giorgia Vaccaro, Rosalind Gittins, Salvatore Ferla, Fabrizio Schifano, Amira Guirguis

**Affiliations:** 1 Department of Clinical, Pharmaceutical and Biological Science, School of Life and Medical Sciences, University of Hertfordshire, Hatfield AL10 9AB, UK; 2 Clinical Department, WDP-Westminster Drug Project, 18 Dartmouth St., London SW1H 9BL, UK; 3 Pharmacy, Medical School, The Grove Extension, Swansea University, Swansea SA2 8PP, UK

**Keywords:** drug checking, drug detection, point-of-care testing, harm reduction, drug-related deaths

## Abstract

Across the world, the interest in point-of-care drug checking as a harm-reduction intervention is growing. This is an attempt to improve intelligence about current drug trends and reduce drug-related morbidity and mortality. In the UK, drug-related harm is increasing exponentially year after year. As such, specialist community treatment services are exploring new methods to improve engagement with people who use drugs (PWUD), who may require support for their problematic drug use. This need has driven the requirement to pilot an on-site, time-responsive, readily available drug-checking service at point-of-support centres. In this study, we piloted the UK’s first Home Office-licensed drug-checking service that was embedded into a community substance-misuse service and had all on-site analysis and harm-reduction interventions led and delivered by pharmacists. We report on the laboratory findings from the associated confirmatory analysis (UHPLC-MS, GC-MS, and ^1^H NMR) to assess the performance of the on-site hand-held Raman spectrometer and outline the challenges of providing real-time analysis of psychoactive substances in a clinical setting. Whilst acknowledging the limitation of the small sample size (*n* = 13), we demonstrate the potential suitability of using this technology for the purposes of screening substances in community-treatment services. Portability of equipment and timeliness of results are important and only very small samples may be provided by people who use the service. The challenges of accurately identifying substances from complex mixtures were equally found with both point-of-care Raman spectroscopy and laboratory confirmatory-analysis techniques. Further studies are required to confirm these findings.

## 1. Introduction

In 2020, the United Nations Office on Drugs and Crime (UNODC) reported substantial increases in global drug use in terms of the number of people who use drugs (PWUD) and the percentage of the population that has developed substance-use disorders (SUD) [1]. Surprisingly, only one in seven people with SUD received some form of treatment [1]. In fact, UNODC projections reported over 269 million PWUD globally, a staggering 5.3% of the population, and postulated a likely exponential rise in SUD as a response to the worldwide COVID-19 outbreak [1]. In its 2022 report, the UNODC reported that 5% of all substance-related deaths are due to drug use, and that drug use accounts for approximately 9% of substance-related disability and premature deaths [2]. Factors influencing this rise in drug use include the dramatic change in the drug supply chain over the past two decades, which has witnessed gross adaptations to achieve increased user accessibility and vendor visibility using social media and the internet, thus leading to easy-to-access alternative “highs” and new psychoactive substances (NPS) through both global and domestic clandestine operations [3]. These developments coupled with shrinking treatment-and-prevention budgets have led to an unprecedented hike in public-health risks and a significantly increased burden to society [4,5]. In the United Kingdom (UK), the drug-treatment sector is now planning to receive notable investment since the societal burden of illicit drug use continues to grow, with a total cost of about GBP 20 billion per year [4,6] and increasing mortality rates. These rates exceeded 50 per million in 2019 [7], increased to 79.5 deaths per million in 2020, and continued to increase by a further 6.2%, reaching 84.4 deaths per million in 2021 [8]. The UK has recently recorded harm from substance use at its highest level for over a decade [8,9,10], with Harm Reduction International confirming similar trends globally [11].

Responding to these challenges is complex and emotive, requiring both public and governmental engagement to deliver improvements in public-health outcomes, as well as being critical to the avoidance of premature drug-related deaths both across the UK and globally [12,13]. A number of recent studies have sought to address these challenges and driven focus towards harm-reduction strategies [14,15,16,17,18,19,20,21] with versatile interventions to reduce the negative impacts from drugs [16,22,23]. These include needle-and-syringe provision, naloxone distribution, opioid-substitution therapy (OST), supervised consumption of OST, psychosocial interventions, and drug checking.

In-field drug checking has recently been documented as a harm-reduction intervention and has been employed predominantly around music festivals and nightclubs [19,24,25,26]. Drug checking enables people who intend to use a psychoactive substance to anonymously check its content prior to consuming it and obtain appropriate and timely advice [27]. Usually, the procedure involves submission of the drug for analysis followed by a verbal consultation, and relies on presumptive colorimetric tests, portable Fourier-transform infrared (FTIR) spectroscopy, or more detailed analysis performed in a remote laboratory [28].

Trials in this field have reported numerous models (e.g., employing spectroscopic and chromatographic techniques as well as presumptive tests) and improved developments in the detection and identification of illicit and novel drugs, although they are often associated with mixed opportunities and challenges [3,29,30]. Portable in-field techniques with their screening ability are usually easy to use and beneficial in providing a low-cost rapid identification of the content of the sample and informing on decisions accordingly (e.g., advice on harm, providing intelligence information, preparing for possible threats from high-risk drugs) [31,32]. However, they may be associated with a high rate of inaccuracies. In contrast, benchtop (e.g., ion mass spectrometry (IMS)) and laboratory-based techniques provide confirmatory and more accurate analyses. Unlike IMS, laboratory-based techniques can be logistically challenging to relay results to individuals in a time-sensitive way, but both require expertise and can be very costly [16].

Remote lab-based services have long been revered as the preferred one-stop diagnostic solution for drug checking, offering high discrimination-power access to advanced analytical techniques that provide greater specificity and broader spectrum analysis [33]. Many laboratories employ variants in liquid chromatography coupled with multiple detection systems that tend to include mass spectrometry (MS) or infra-red (IR) detectors. High-standard techniques include ultra-high-performance liquid chromatography–mass spectrometry (UHPLC-MS), liquid chromatography–mass spectrometry (LC-MS), and liquid chromatography–infrared detection (LC-IRD), with the gold standard long considered to be gas chromatography–mass spectrometry (GC-MS). Each test offers infinite broad-spectrum analysis of both known and unknown compounds [15]. These techniques are destructive, and identification of the unknowns largely relies on software capabilities, built-in libraries and algorithms, and the availability of reference standards.

Key in-field techniques include colorimetric presumptive tests (spot tests), thin-layer chromatography (TLC), and self-testing strips, with spot tests being the most widely applied techniques due to their selectivity in identifying functional groups or a structural backbone [16,31,32,34]. Although these tests can be undertaken with minimum training, they are largely limited by their increased specificity and selectivity, leading to a high rate of cross-reactivity and false positives when compared with laboratory-based analyses, along with reduced discriminating capability [33,35]. In contrast, vibrational techniques such as FTIR testing in a laboratory offers a complex, broad-spectrum analysis capable of delivering reliable and accurate results, rapid analysis times, and the ability to test both solid- and liquid-state samples. This non-invasive technique preserves the integrity of the sample, which is a significant advantage [36]. Low sample concentration and high sensitivity are limiting factors, as the reliance on IR analysis is highly dependent on the abundance of the compound of interest, with detection limits for fentanyl as low as 83% sensitivity (“ability to detect true positives”) and 90% specificity (“ability to detect true negatives”) recorded [34].

Vibrational techniques, predominantly infrared techniques, have been attractive tools for on-site detection of drugs due to their availability in handheld versions [37]. FTIR is considered a preferred forensic analytical tool that can be used in the field due to its high potential in discriminating between drug isomers, which has a significant impact on the identification of the so called “legal high” analogues [36,38]. However, FTIR performance is limited by the presence of high amounts of cutting agents and minute amounts of active agents. Near-infrared (NIR) performance is also limited by the presence of moisture and is susceptible to the physical characteristics of the analytes of interest as well as the presence of cutting agents. Raman spectroscopy is known to be complementary to infrared spectroscopy based on the law of mutual exclusion [39]. Raman spectroscopy has shown advantages over IR techniques, demonstrating a proof-of-concept in its discriminatory power for the identification of the active substance(s) as opposed to the cutting agents [40]. It also allows for non-destructive through-package analysis with no sample preparation required and minimum interference from moisture and physical characteristics of the substance(s) of interest [39]. Raman strength also relates to its ability to produce good-quality spectra from very small sample sizes, e.g., containing a few powder particles. Its main limitations related to dark drug samples, which absorb high amounts of laser energy and burn (even with a low-energy long laser λ_ex_ such as 1064 nm), and samples containing fluorescent adulterants, which may produce fluorescent signals that swamp the weaker Raman signals.

The Scientific Working Group for the Analysis of Seized Drugs (SWGDRUG) categorised Raman analysis as a Category A analytical technique alongside other Category A techniques, such as infrared spectroscopy, mass spectrometry, nuclear-magnetic-resonance spectroscopy, and X-ray diffractometry. Category A techniques have high discriminatory power [33]. However, the discriminatory power may be compromised by the challenging nature of some mixtures. The Group recommended that Category A techniques should be coupled by at least one Category B (e.g., gas chromatography) or Category C technique (e.g., ultraviolet spectroscopy) [33]. The UNODC confirmed these recommendations, highlighting that on-site handheld Raman detection can be used as a screening tool that should be coupled with laboratory-based confirmatory analysis [41]. Raman spectroscopy has been trialled for the detection of traditional drugs of abuse, such as 3,4-methylenedioxy-N-methylamphetamine (MDMA), cocaine and heroin [42], and psychoactive substances [43,44,45,46]. Raman spectroscopy plays an important role as a diagnostic tool in other applications such as cancer and disease-biomarker detection [47,48].

In drug checking, FTIR requires specific criteria to be met in terms of effective venue siting due to the equipment relying upon a firm, vibration-free environment [27].

Drug-checking services carry a deep-rooted stigmatisation in terms of criminality and morality [21]. Public engagement in this new era of drug awareness was tested in a netnographic Twitter analysis aimed toward capturing a snapshot of current public perception regarding the provision of drug-checking services [3]. This study highlighted an even split in the eyes of the public between support for and denial of the need for drug-checking and harm-reduction services. Although globally well received, much of the UK perspective was not captured by this research, in part due to the novelty of the intervention and due to the indicative paradox of public perception being stranded between the premise of being emotively controversial and undeniably encouraging [16,28,49].

To date, there is limited current research available detailing the combination of in-field real-time drug-checking and harm-reduction measures being delivered to PWUD in a clinical setting, with several studies documenting limited universal government support for this type of research [17,25,29,50,51]. Encouragement for further research engaging with the public and the government led to the development of the UK’s first Home Office-licensed drug-checking service embedded within a community substance-misuse service (SMS), which was also pharmacist led [3]. This was a bespoke anonymous service with on-site drug analysis coupled with harm-reduction interventions conducted by pharmacists based on multidisciplinary decisions.

In this study, we aim to assess the performance of the associated hand-held Raman spectrometer that was used in this novel service [3]. We present the confirmatory analytical characterisation of the drug samples for comparison and the challenges faced when providing a real-time analysis of psychoactive substances in a clinical setting.

## 2. Materials and Methods

Timeframe: The pilot service operated for four days between 22 February and 15 March 2019.

Settings: Drug samples were handed over by anonymous PWUD, with the samples checked on Addaction’s community-service site in Weston-Super-Mare in North Somerset using a handheld Raman spectrophotometer (Progeny^TM^) [3]. The samples were then transported to a remote laboratory (University of Hertfordshire) for confirmatory characterisation using UHPLC-MS and GC-MS and proton nuclear magnetic resonance (^1^H NMR). The transport of the samples followed the UK legislation and Home Office requirements for the lawful possession and supply of controlled drugs between sites and complied with associated record-keeping regulations for controlled substances. Information on the site selection, the patient and public involvement in the study design, risk assessments, participating stakeholders, compliance with legislative requirements, staff training, client recruitment, and cascading alerts are detailed in Guirguis et al. (2020) [3].

Samples: Street samples (Table 1) were supplied for analysis courtesy of people accessing the service in North Somerset, UK, under an associated Home Office licence.

Chemicals and Reagents: Reference standards for diazepam, caffeine, and cocaine were sourced from Sigma Aldrich (Cambridge, UK). Reference standards for etizolam, flubromazepam, pyrazolam, and the synthetic cannabinoid THJ018 were obtained from Chiron AS, (Trondheim, Norway). Deuterated methanol d-4 was sourced from Cambridge Laboratories (Cambridge, CA, USA), and UHPLC-grade methanol (MeOH) and formic acid 98–100% were obtained from Fisher Scientific (Loughborough, UK). Millipore water was produced in house using a Merck Millipore 0.22 µm filtration system (Darmstadt, Germany) employing a Millipake Express 20 filter system, providing water quality at 15.2 MΩ. cm at 25 °C.

Sample Mass Recording: In line with the Analytical Procedures and Method Validation for Drugs and Biologics 2015 guidelines and Home Office requirements, each sample was weighed pre- and post-sample working-stock preparation using a Mettler Toledo Balance operated inside a BIGNEAT F3-XIT enclosed safety cabinet (Hampshire, UK), which was capable of measuring 0.01 mg up to 220 g. Samples were also weighed pre- and post-GC-MS, UHPLC-MS, and NMR analysis to record evidence of experimental/analytical/sample-transfer loss using the same Mettler Toledo Balance. All preparations and analyses were performed at an ambient room temperature of ca. 26 °C.

Reference Standard and Sample Preparation: Reference standards of diazepam, etizolam, flubromazepam, pyrazolam, cocaine, caffeine, and THJ-018 were prepared for analysis under identical experimental conditions as the street samples, diluting 2 mg standard material to 25 mL with UHPLC Grade MeOH, yielding a final working-stock solution of 80 µg/mL. Due to the limited street-sample size, a method of analysis was developed to produce a single working-stock solution for each sample. This was performed by macerating each sample in a sterile, acid-washed glass vial and adding between 1 and 3 mL of UHPLC Grade MeOH. The mixture was then vortexed at level 4 (Vortex Genie 2, Scientific Industries, Bohemia, NY, USA) for 2 min, then sonicated for 5 min to maximise the active-ingredient-extraction process. The resulting supernatant was finally filtered through a 13 mm 0.22 µm PTFE syringe filter (Thermo Fisher Scientific, Hemel Hempstead, UK) directly into a Waters 12 × 32 mm UHPLC-MS glass chromatography vial (Waters, Borehamwood, UK) ready for analysis. Post filtration, any remaining physical materials were re-sealed and retained within their original evidence bag for future analysis or to be denatured in line with UK legislation. Given the limited sample size (ranging from a few powder particles and powder traces to a full tablet or a few milligrams of powder), each working-stock solution was extracted from UHPLC vials into GC-MS vials for GC-MS analysis using sterile procedures. Preparation for NMR analysis required each sample to be evaporated to dryness with nitrogen within their GC-MS vials using a Techne Sample Concentrator for 30 min. Samples were then re-solubilised with 1.5 mL Methanol-d4, followed by 2 min in a vortex and 5 min sonication to maximise the dissolution process. Samples were individually transferred to a Wilmad 5 mm thin-wall precision NMR sample tube (Sigma Aldrich, Haverhill, UK) for analysis.

**Figure 1:**
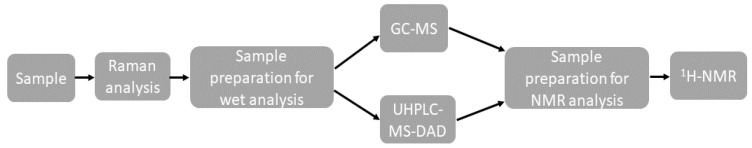
Summary of the analytical process.

A summary of the analytical process is shown in Figure 1.

Experimental-Blank Preparation: Blanks provided for analysis were treated under the same conditions as the samples and reference standards by 0.22 µm PTFE filtering HPLC-grade MeOH followed by 2 min vortex and 5 min ultrasonication.

Analytical Conditions: In line with the Analytical Procedures and Methods Validation for Drugs and Biologics 2015 guidelines, each sample was prepared and analysed in triplicate, with double blank measurements between consecutive runs to assess any potential carryover or interferences. All preparation and analyses were performed at an ambient room temperature of ca. 22 °C.

Gas Chromatography–Mass Spectrometry (GC-MS): Gas chromatography–electron ionisation–mass spectrometry (GC-EI-MS) analysis was performed to identify unknown compounds present within unspecified street-drug samples. The method was adopted from Assi et al., (2015). Analysis was performed using a Varian 450GC equipped with a Varian 8400 Autosampler and Varian 240-MS from Agilent Technologies (Didcot, UK). Spectral/MS data were processed using a Varian MS Workstation installed with Varian MS Data Review Software, version 6.9.2. Samples were analysed using an electron-ionisation (EI) target total ion chromatogram (TIC): 20,000 counts, over a scan range of *m*/*z* 40 to *m*/*z* 700. An Agilent Technologies column (30 m × 0.25 mm × 0.25 µm) was coated with a 50:50 coating of phenyl:methylpolysiloxane along with helium-gas mobile phase at a flow rate of 1 mL/min^−1^. The EFC injector was held at 275 °C and was used in split mode (50:1) for every sample to analyse triplicate 1 µL injections. Column temperature and hold parameters: a 50 °C holding temperature was ramped up to 150 °C and held for 3 min; this was then ramped up to 250 °C and further held for 1.33 min, with the final stage increasing to 310 °C and held for 6 min, giving a total run time per sample of 20 min.

Ultra-High-Pressure Liquid Chromatography–Mass Spectrometry with Diode-Array Detector (UHPLC-MS-DAD): A Waters Acquity CM UHPLC-MS (MS) equipped with a Waters Autosampler, an Acquity PDA Diode Array Detector, a Waters Acquity QDa Mass Spectrometer, and MassLynx V4.2 SCN976 software was used to operate the system and process all data. A Phenominex Kinetex C18 100Å Column, 100 × 2.1 mm i.d. and 2.6 µm pore size, was purchased from Phenominex (Macclesfield, UK). Mobile phases A and B comprised 0.1% formic acid in water and 0.1% formic acid in methanol, respectively. Each mobile phase was filtered using a 022 µm Sinter filter system. A gradient method was employed with an initial phase of 50:50 over 0.5 min; a linear gradient of 10:90 was driven through 0.5 to 3.5 min, equilibrating to 50:50 at 4.0 min until complete, with a total run time of 6 min. Column temperature remained constant at a room temperature of ca. 28 °C. The injection volume was 0.2 µL and all injections were done in triplicate, with a double blank injection between all samples. Spectral MS data of unknown compounds were produced using the PDA at 20 sampling points per s, and PDA was set to 1.2 nm resolution across 190–400 nm. Electrospray (ESI) at ca. 3–5 kV results in ions contained in the aerosol droplets that are protonated and detected in the form of [M + H]^+^ in positive-ion mode. This results in identifiable fragments from which the molecule’s identity can be determined.

Nuclear Magnetic Resonance (NMR): ^1^H and COSY NMR spectroscopy were performed on a Jeol ECA600 spectrometer equipped with an HCN probe, with the spectral analysis processed using Delta 4 software. Identification was carried out using COSY NMR (relaxation delay–pulse acquisition). Typical acquisition parameters were as follows: X_width 6.25 [us], X_acquisition time 1.45 s, X_angle 45°, relaxation delay 4 s, and 64 scans. Assignment of signals was achieved by comparison with known spectra of pure compounds.

All MS and NMR spectra obtained were initially compared to tested reference materials; additional electronic resources for reference included the Perkin Elmer online predictive NMR resource (Perkin Elmer Chemdraw, Beaconsfield, UK), SWGDrug online resources (Woodbridge, VA, USA), Cayman Chemical online resources (Ann Arbor, MI, USA), npsdiscovery.org (Willow Grove, PA, USA), J Wiley & Sons (Medford, MA, USA), nmrdb.org (Cali, Columbia), MolBase online resources (Shanghai, China), Mass Bank of North America online resources (Davis, CA, USA), Royal Society of Chemistry ChemSpider online resources (Cambridge, UK), National Library of Medicine online resources (Bethesda, MD, USA), and Ceondo GmbH Chemio online resources (Gelsenkirchen, Germany). Tertiary analysis was completed using in-house spectral/MS library information that was developed and supplied courtesy of Dr. Amira Guirguis and the University of Hertfordshire (Hatfield, UK).

Raman Spectroscopy: A Rigaku Progeny^TM^ (SciMed Ltd, Stockport, UK.) instrument with an Nd:YAG (neodymium-doped yttrium-aluminium garnet) laser λ_ex_ of 1064 nm (laser-output power range of 30–490 mW at the source; laser spot diameter of 20 µm) was employed for the on-site checking of drug samples provided by individuals accessing the service. The instrument had a spectral resolution of 8–11 cm^−1^, a spectral range of 200–2500 cm^−1^, a numerical aperture of 0.25; a transmission-volume-phase (VPG) grating with 818 lines/mm, and a TE cooled InGaAs 512 pixel detector. The exposure time was adjustable over the range of 5 ms to 30 s. The operational and analysis software was RRT Progeny software version 0.001-26 140521. The instrument was equipped with the wavelet correlation coefficient (WCC) and the Rigaku-mixture algorithm with multiple data-export formats, including PDF, .xml, and .txt. Based on the research of the senior/corresponding author of this article, different laser wavelengths were evaluated for the detection of psychoactive substances in drug mixtures [52]. The study compared the 785 and 1064 nm excitation wavelengths aiming at the detection of active psychoactive substances in 60 products purchased over the internet. Results from this study suggested an improved identification of the psychoactive agents and a better discrimination against cutting agents in the complex mixtures using the 1064 nm excitation by 48%. It is stipulated that this was potentially because of the longer wavelength’s (λ_ex_ = 1064 nm) inherent lower energy, which resulted in reduced fluorescence, amplified Raman signals, and enhanced detection of the psychoactive substances. These findings support the use of the 1064 nm λ_ex_ in this study for potential enhanced detection of the active ingredients.

Four methods were used to collect Raman spectra based on the substances’ nature: method A (2000 ms exposure time; 490 mW laser power; 10 averages), method B (2000 ms exposure time; 200 mW laser power; 10 averages), method C (2000 ms exposure time; 100 mW laser power; 10 averages), and method D (2000–5 ms exposure time; 50 mW–1 mW laser power; 10 averages). All samples were initially analysed using method A, but methods B and C were employed for samples that were burned or were prone to burning from high laser power (i.e., coloured samples). Method D was developed to collect Raman signals from challenging samples that exhibited intense fluorescent background and/or were burned with method C by adopting an iterative approach to reducing both the laser power and exposure time. All methods used a built-in baseline correction function for each measurement. The instrument was calibrated each day immediately before analysis using a benzonitrile reference standard. All samples were analysed directly through glass vials, plastic bags, or cling film. Obtained spectra were automatically compared to the on-board reference library and a percentage correlation was reported using the built-in Rigaku-mixtures algorithm (RMA). All spectra were individually inspected and compared to reference spectra for confirmation of findings.

## 3. Results and Discussion

**Figure 2:**
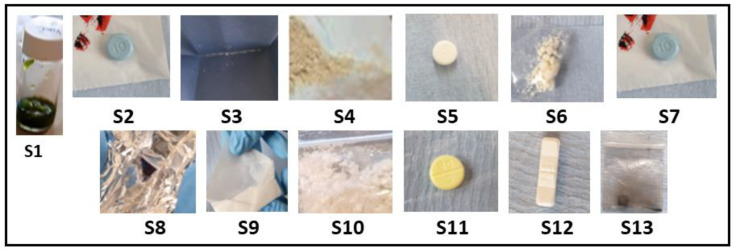
Images of each sample surrendered for analysis at the drug-checking clinic; each sample was weighed and described prior to test (Table 1). Please note sample S1, a herbal material, was suspended in methanol prior to the image being taken.

The pilot service operated over four days. Samples were initially analysed on-site using a handheld Raman spectrophotometer (Progeny^TM^) and then sent to the lab for confirmatory characterisation at the University of Hertfordshire laboratories. Thirteen samples were submitted, consisting of multiple drug classes and forms—stimulants (*n* = 4), synthetic cannabinoids (*n* = 1), opioids (*n* = 1), depressants (*n* = 5), empathogens (*n* = 1), and psychedelics (*n* = 1)—and assigned to a unique sample code (S1–13). All surrendered samples were photographed at the point of amnesty (Figure 2).

The process of identifying the content in the samples is explained in detail for the first set of samples only to avoid repetition.

### 3.1. Stimulants

**Figure 3:**
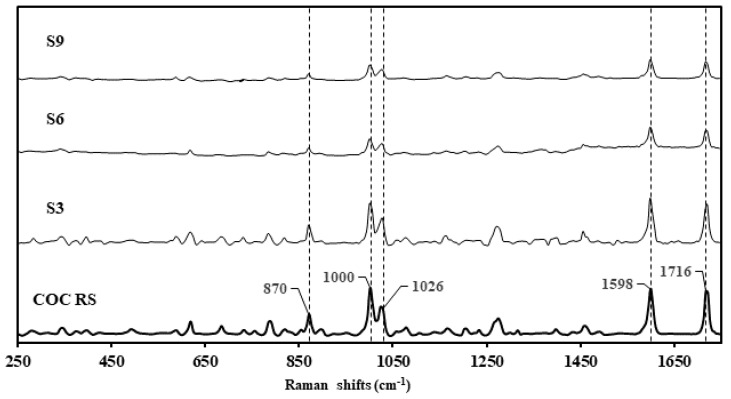
Raman spectra of cocaine reference standard (COC RS) stacked against the surrendered samples, S3, S6 and S9, declared as cocaine samples by the clients.

Samples S3, S6, and S9 were presented in non-similar packaging, with the physical compound being similar in appearance: a granular, hygroscopic white powder (Figure 2, Table 1). S10 presented dissimilar to S3, S6, and S9 as a clumpy, flocky powder. Using the Rigaku-mixture algorithm, the handheld Raman analysis for S3, S6, and S9 was definitive in terms of signal intensity and probability of matching (CC > 96%) correlating to cocaine hydrochloride (Figure 3). See Raman peak assignments in Supplementary Materials.

Raman analysis for S10 was performed through packaging for sample preservation and with improved signal response. the analysis confirmed the content as possible caffeine and creatine, which are both common cutting agents (CC > 85%).

**Figure 4:**
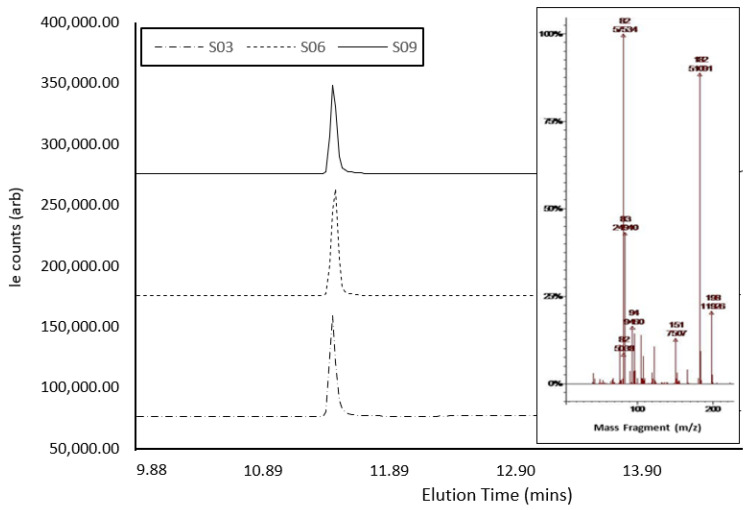
GCMS spectra of cocaine samples S3, S6, and S9, showing elution times and peak size, with the MS-spectra inlay showing abundant molecular ions at *m*/*z* 182 and 82.

Confirmatory GC-MS analysis (Figure 4) showed abundant molecular ions at *m*/*z* 182 and 82, which are consistent as dominant precursors for cocaine in the literature [42]. The active compound within S3, S6, and S9 eluted at 11.041 min, 11.024 min, and 11.021 min, respectively (Table 2). The three samples presented as hygroscopic powder, as expected, with cocaine hydrochloride (339.8 g/mol^−1^), a result of the presence of a salt under GCMS analysis. However, GCMS analysis of S6 and S9 indicated a mass for the compound as ca. 303 g/mol^−1^, which could correlate with the cocaine base (MW of 303.35 gmol^−1^) [53,54]. The GCMS analysis of S10 confirmed the presence of caffeine in the sample, which presented a single peak at ca. 8.22 min, with a molecular ion at *m*/*z* 194. Fragmentation ions and associated NIST library matches also indicated the presence of caffeine. A comparison between sample ion-fragmentation patterns confirmed the presence of caffeine as the single compound within the sample [55]. Creatine, which does not dissolve in MeOH, was possibly isolated and hence was not identified with GC-MS [52]. Spectral traces of S3, S6, and S9 demonstrated near-identical instrument response under GC-MS interrogation, even though each sample was collected in the field on different days and from different individuals, showing the potential for these samples to have been produced from the same batch. There was a notable difference in signal intensity across the three samples, possibly indicating slight variations in cocaine concentration.

**Figure 5:**
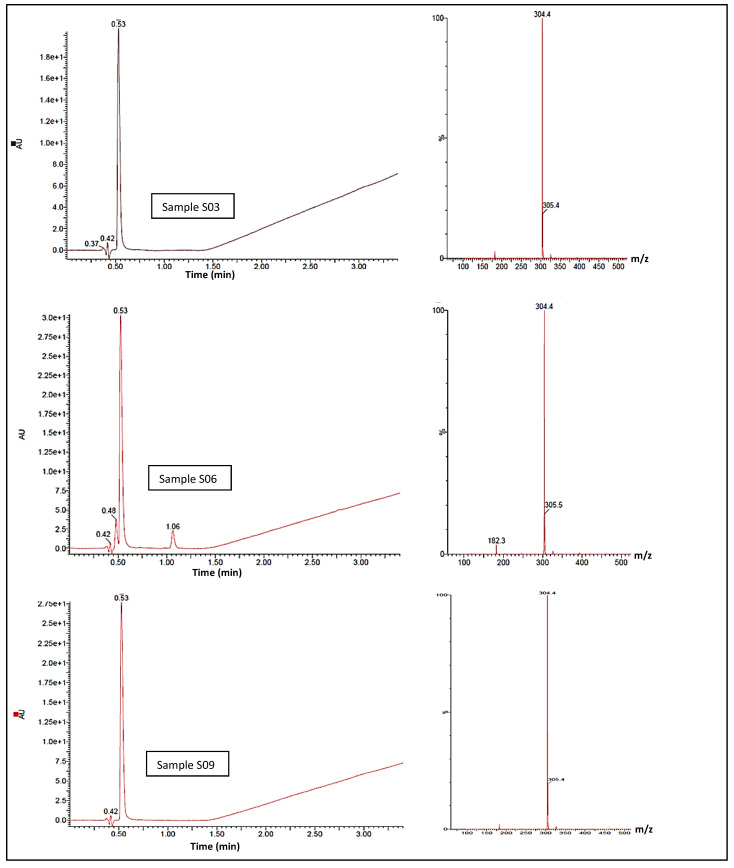
UHPLC-MS spectra showing cocaine elution time circa 0.53 minutes, and cocaine elution signal intensity, for samples S03, S06 and S09 (left). MS-spectra (right) for the samesamples showing abundant molecular ions at *m*/*z* 304.4 and 305.4, consistent with cocaine.

The UHPLC-MS confirmatory analysis of S3, S6, and S9 (Figure 5) resulted in all samples exhibiting a single chromophore response at ca. 0.53 min; this pair of lone peaks was compared to and concurred with the instrument response when cocaine reference standards were analysed under identical conditions. The ES+ spectra for both samples exhibited molecular ions at *m*/*z* 304.5 and 305.4, illustrating the ^13^C isotope version of *m*/*z* 304.5. There was no evidence of an MH+ peak with an expected 3:1 ratio within the MS, indicating the lack of a chlorine atom in either sample, which correlates to the cocaine base (MW 303.35 gmol^−1^). The diode-array-detector (DAD) response confirmed a single compound contained within both S3 and S9. In terms of peak retention time and λmax, these were also indicative of a cocaine base when compared to tested cocaine reference standards. MS spectra did not indicate that any dimerisation occurred, although S3 showed a final ion with a nominal mass at *m*/*z* 326.4 and S9 showed a final ion with a nominal mass at *m*/*z* 326.2; this was likely due to the presence of a sodium adduct MNa+, a by-product of the UHPLC-MS process. With adjustments for the ES+, the compound of interest in both samples was likely to be a mass of ca. 303.4 g/mol^−1^, concurring with the GCMS result. Under the same conditions, the DAD response showed a second compound eluting at 1.064 min, potentially indicating the presence of a second unidentified compound within the S6 with a molecular ion at *m*/*z* 138.4 at ca. 1.064 min. S10 was subjected to identical experimental conditions, and the DAD response showed a single peak response at ca. 0.57 min. MS for this peak was compared against the ES+ spectra and was shown to exhibit a protonated molecular ion of *m*/*z* 195.4, which is indicative of caffeine (MW 194.19 gmol^−1^) with a λmax of 272.8 nm.

**Figure 6:**
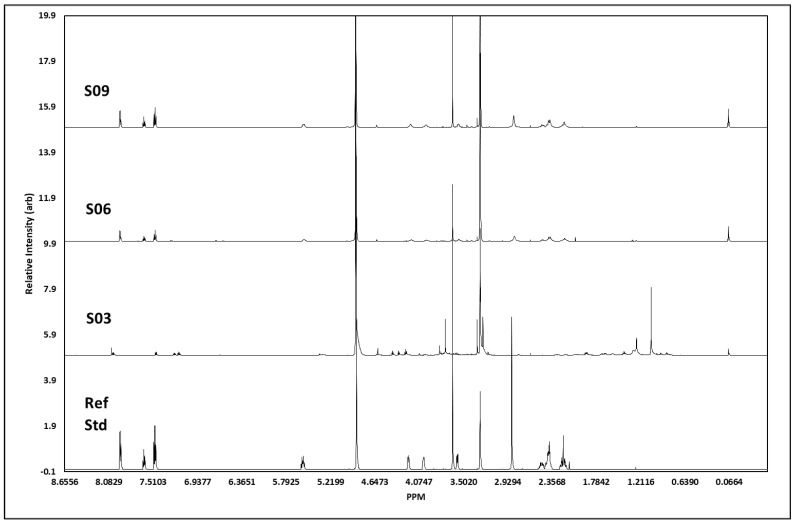
^1^H NMR spectral data. Figure shows spectral trace for each cocaine sample, S03, S06 and S09, with a pharmaceutical reference standard (Ref Std) below. Spectra shows distinct areas of singlet and multiplet signatures, concurrent with those visible in the reference standard, as expected with ^1^H NMR. The additional noise in the surrendered sample spectra is indicative of street samples of cocaine, which has been cut with unknown agents.

The ^1^H NMR analysis (for all J-coupling reports, see Table 2) of S3 showed all the proton signals we would expect to see from cocaine (Figure 6). In addition, there were some other peaks, presumably from other minor components. The peaks at 7.47–7.50 ppm, 7.61–7.64 ppm, and 7.93–7.94 ppm showed signals characteristic of the aromatic protons in the cocaine molecule, with chemical shift, integrations, and splitting patterns consistent with that structure. The signal at 5.56 ppm related to the proton on the cycloheptane ring closest to the deshielding oxygen atom, whereas the two signals at 4.18 ppm and 3.98 ppm were for the two single protons on the carbon atoms bridging to the nitrogen atom, which is responsible for deshielding the methyne proton signals and broadening the peaks so that they are seen as singlets. The other methyne proton on the cycloheptane ring, next to the carbonyl carbon, was seen as a broad singlet 3.57 ppm. The two methyl-proton signals, each with three protons, showed as singlets at 3.64 ppm (the methyl attached to the oxygen atom of the carboxylate group), and the 2.84 ppm was from methyl protons attached to nitrogen, only showing as 2.4 protons but broad, as would be expected. The remaining six protons resulting from the methylene groups in the cycloheptane ring showed as two overlapping multiplets between 2.4 and 2.5 ppm containing four protons and a multiplet at 2.2 ppm from the two protons closest to the methine proton at 5.6 ppm. The total number of protons generated through software integration was 21, concurring with the expected proton count for cocaine. S6 showed a high number of protons we would expect to see from cocaine; however, not all were visible, and the spectra showed additional peaks, presumably from additional adulterants. Unlike S3, the two methyl-proton signals, each with three protons at ca. 3.64 ppm, were not visible; however, the signal at 2.85 ppm from the methyl attached to the nitrogen atom remained. The remaining six protons resulting from the methylene groups in the cycloheptane ring showed as two overlapping multiplets between 2.38 and 2.5 ppm containing four protons and a multiplet at 2.19 ppm from the two protons closest to the methine proton at 5.6 ppm. Unlike S3, the peaks at 3.3 ppm and 3.56 ppm were not picked up in the J-coupling report; the spectrum integration was 0.16 and 0.65 protons, respectively, with the first peak on the side of the methanol peak. Evidence suggests the concentration of S6 was weaker than that of S3, which would explain the lack of certain associated peaks we would expect to assign. S9 showed all the proton signals we would expect to see from cocaine. Although the aromatic region at 7.47 ppm and 7.65 ppm showed almost the exact same profile between S3, S6, S9, and the cocaine reference standard, the response at ca. 7.95 ppm differed between S3 and S6/S9; this is possibly due to either atomic interference of adulterants within the sample or due to differences in the concentration between each sample.

Similarly, the three samples and cocaine reference standard appeared to share a similar chemical profile; however, S3 also indicated the probability of additional materials within the sample, resulting in additional proton peaks between 1.4 and 1.95 ppm in the aliphatic region. Further chemical-shift changes were visible within the splitting region, plus some additional compound(s) resulting in proton peaks ca. 4.3–4.41 ppm in S3. J-coupling reports evidenced the presence of cocaine within the samples, confirmed by Raman and GCMS analysis; however, any additional adulterants/active pharmaceutical ingredients (APIs) were not characterised within this study, primarily due to the sampling process and quantity of sample retained in-field. There was a strong visual correlation in spectral data between S6 and S9, which could infer these samples to be of the same parent batch. Chemical shifts and peak profiles for both samples were identical, as were the peak intensities and duration. In contrast, S3 appeared to contain additional impurities that added several profiles inconsistent with the other samples and the reference standard and resulted in a chemical shift downfield. The J-coupling report (Table 2) for S10 confirmed the identification of all four peaks expected for caffeine within the sample, namely, the proton on the imidazole ring at 7.85 ppm, along with all three singlets at 3.95, 3.51, and 3.33 ppm, each containing three protons from the three methyl groups attached to nitrogen atoms, resulting in three peaks showing three protons at each peak. However, in addition to these known signals, there were also two major signals at 3.83 ppm and 3.00 ppm that appeared to represent a non-caffeine component. Therefore, we can identify caffeine as a major compound within this sample with certainty, but the sample also contained additional components. Comparison of the spectra from S10 and the caffeine reference standard showed near-perfect correlation in caffeine peaks assigned to the sample, but with the inclusion of unconfirmed adulterant(s) material at 7.5–7.35 ppm, 4.31–4.4 ppm, and 2.99 ppm.

### 3.2. Synthetic Cannabinoid-Receptor Agonists (SCRAs)

A herbal “spice” sample was submitted for analysis. The Raman analysis correlated to taurine. This could not be confirmed and was deemed to be a false-positive result. The sample was soaked in MeOH, and the resulting solution was analysed again, but still matched to taurine (CC > 76%). Upon inspection, the spectrum showed weak Raman peaks hindered by a significant fluorescence signal. Wet analysis using GC-MS, UHPLC-MS, and ^1^H NMR could not confirm the presence of tetrahydrocannabinol (THC) or any backbone similar to traditional SCRAs. Previous studies showed that the analysis of herbal SCRAs in drug-checking services is particularly challenging [56].

### 3.3. Depressants

The Raman analysis of S2 and S7 were presented in the same way by different individuals reportedly from different nearby cities, and the samples were identical in appearance and markings, unlike S5, S11, and S12, which showed no similar physical characteristics with the other samples. Each showed a uniform, smooth finish, as expected with a genuine pharmaceutical product. Physically identical S2 and S7 showed a glossy outer blue coating and a high content of cutting agents, which are typical characteristics that have been shown to hinder Raman detection. Raman response for S2 indicated a possible starch, talc, and carboxy-methylcellulose content but did not identify any active pharmaceutical compounds, which were possibly present in very small concentrations. Solutions made from these tablets could not identify the presence of an active ingredient. The Raman response for S7 suggested a content including talc once again, potentially including 5-iodo-2-aminoindane and diazepam. Whereas the presence of talc, or magnesium silicate (MgSiO_3_), and diazepam would be commonplace in the production of pharmaceutically produced benzodiazepine tablets, 5-iodo-2-aminoindane is being sold as a new psychoactive substance. S5, S11, and S12 were analysed directly using the handheld Raman instrument. S5 reported a high content of lactose and showed increased fluorescence signals, resulting in possible false positives for amphetamine analogues. S11 analysis confirmed the possible content of α-lactose monohydrate, whereas S12 analysis confirmed the presence of microcrystalline cellulose, another common bulking element used in the drug-manufacturing process. The physical characteristics of S11, including markings, size, and colour, were highly comparable to the genuine pharmaceutical product, diazepam tablet BP 5 mg, produced by Accord-UK Ltd. The genuine product is manufactured with a confirmed excipient list of 152.00 mg lactose, plus magnesium stearate, maize starch, stearic acid, and E104. However, only the content of lactose in the sample was confirmed by Raman, not the expected API. Similarly, S12 also appeared to have the identical physical characteristics as a genuine pharmaceutical product, Xanax^®^, produced by Pfizer U.S. Pharmaceuticals Group. The genuine product is marketed and sold as a benzodiazepine with 2 mg of the active ingredient, alprazolam, with a confirmed excipient list that includes microcrystalline cellulose, lactose, magnesium stearate, maize starch, colloidal anhydrous silica, and docusate sodium with sodium benzoate. Onsite, the content of microcrystalline lactose was confirmed by Raman but not the API.

The GC-MS analysis of S2 showed a single peak of interest in the sample, with a retention time of 16.579 min and reporting a molecular ion of *m*/*z* 343. Interrogation of the fragmentation patterns available for this peak indicated the presence of a single compound within the sample, strongly indicating the presence of etizolam. S7 resulted in a similar chromatogram, showing a single peak of interest at 16.550 min and reporting a molecular ion at *m*/*z* 343. Interrogation of the fragmentation patterns available for this peak also indicated the presence of the etizolam compound. Comparison of the two samples showed a distinct similarity in terms of peak retention time and fragment pattern; however, the peak profile for both samples was dissimilar. As this occurred in the laboratory with the samples being treated with identical extraction methods, we would be fair to assume the sample concentrations were not alike, therefore indicating the samples may have been clandestine compounds. This contradicts the assumption that the markings reported on the tablet were indicative of the genuine anti-anxiolytic medication originally manufactured by Watson Pharmaceuticals Inc., now Actavis, Inc. (Weston, FL, USA). The presented sample was uniform in appearance, with crisp and clear markings and a tablet diameter, all consistent with the genuine pharmaceutical product. However, the active ingredient, diazepam (MW 284.743 gmol^−1^), was not identified through Raman analysis or GC-MS, which would infer that the API was etizolam.

Sample S5 showed a single peak of interest at 12.66 min, reporting a primary molecular-fragment ion at *m*/*z* 275 and a molecular ion at m/z 304. Interrogation of the fragmentation patterns available for this peak indicated the likely presence of a single compound (Table 2), with a 63.1% probable NIST library match for the compound delorazepam (MW 305.1 gmol^−1^). Literature confirms the primary fragment ion of delorazepam under GCMs as *m*/*z* 275 [57]. Delorazepam, an Italian-licensed anxiolytic product, is recorded on the list of benzodiazepines controlled under the Misuse of Drugs Act 1971 and Misuse of Drugs Regulations 2001 and is not licensed for dispensing in the UK. Analytical data for delorazepam are not widely available, and a comprehensive Raman library could not conform the presence of this in the field, but rather merely the bulking agents; however, GC-MS analysis did report the presence of a single compound within the sample, showing a primary molecular fragment at *m*/*z* 275 and parent ion of *m*/*z* 304, concurrent with Cayman Chemicals representation, which reported a GC-MS monograph showing distinct fragmentation patterns for delorazepam; all fragmentation common between S5 and the published literature are available in Table 2. This illustrates a strong positive correlation between the fragmentation patterns recorded in published data and S5.

Analysis of S11 resulted in the identification of a single peak of interest at 12.84 min, reporting a single active compound with a parent ion at *m*/*z* 284, and fragmentation patterns presented in Table 2, indicating a small probability of the presence of diazepam and that this may be a genuine pharmaceutical product, with Raman confirming the correct excipients and GC-MS identifying the correct parent ion. S12 proved to be a far more complex compound than originally anticipated, with GC-MS spectra showing multiple peaks of interest against the methanol blank, reporting three peaks of interest at 11.03 min, 11.70 min, and 12.30 min. It is considered that S12 may potentially have contained three separate structures within the sample. The genuine pharmaceutical product Xanax^®^ contains a single active pharmaceutical ingredient, alprazolam (MW 308.08 gmol^−1^), yet evidence from GC-MS of this sample suggests each of the three active compounds had a non-similar ion-fragmentation fingerprint. NIST library matches were all significant, showing a major affinity to promethazine (MW 284.13 gmol^−1^) at ca. 11.70 min. Although this does not reflect the pharmaceutical compound associated with Xanax^®^, promethazine (as confirmed by the parent ion 287 *m*/*z* at 12.30 min), a sedative antihistamine, is documented as having central depressant effects that are increased in the presence of benzodiazepines. LGC/Eurofins (2019) reported that a vast percentage of seized fake Xanax^®^ tablets (*n* = 915) did not contain alprazolam; rather, 474 tablets contained etizolam (MW 342.07 gmol^−1^), 266 tablets contained clonazepam (MW 315.7 gmol^−1^), 173 tablets contained a mixture of etizolam and diazepam (MW 284.743 gmol^−1^) in the same tablet, and 2 tablets contained no active ingredient [58]. Interpretation of the MS showed a peak ca. 11.70 min at a mass of ca. 284 gmol^−1^, which may indicate the potential presence of promethazine.

The UPLC-MS and ^1^H NMR analyses of both S2 and S7 confirmed the presence of etizolam and confirmed the presence of diazepam in S11. In contrast, the analysis of S5 could not confirm the presence of delorazepam, possibly due to the low concentration of the active drug in the formulated tablet. The analysis of S12 was also inconclusive possibly by all techniques, with the exception of GC-MS. This is possibly due to the high concentration of cutting agents and low promethazine concentration. The NMR analysis of S5 and S12 showed a very complex array of proton signals throughout the aromatic, splitting, and aliphatic regions, as may be expected from an illicit compound; however, due to the complexity of the peak formations across these signature regions, it was not possible to determine any specific structures within the compound. Peaks at ca. 6.70 to 7.50 ppm showed a range of singlet and multiplet activity that is characteristic of aromatic protons, consistent with what would be expected from illicit drugs comprising multiple aromatic-ring structures. The resulting spectra were resolute, demonstrating a clean process and product, although the presence of such complex multiplicity gives rise to the theory that this sample comprised more than one active compound. With S12, this supports the GCMS results somewhat in terms of the low-percentage NIST library match against promethazine, as the presence of more than one compound with a similar fingerprint would inhibit the accuracy of this process. Complex peak signals were also present in the aliphatic region, rendering the identification of these peaks impossible at this stage; thus, the expected compound could not be fully characterised by NMR alone. It is evident that this sample was a complex compound containing at least two active ingredients. It is evident that in the mixture of compounds, the spectra did not confirm several peaks that would be expected within promethazine.

### 3.4. Other

In this set of samples (i.e., S13, S8, and S4), the identity of S13 was not known by the client. The Raman analysis of the black half of a tablet was inconclusive. Analysis of dark samples is typically unsuccessful with Raman spectroscopy, as it leads to absorption of laser energy and burning of the sample.

The GC-MS analysis of S13 showed a major peak of interest ca. 5.52 min, reporting a 95.2% match for N-methyl-3,4-methylenedioxyamphetamine (MDMA) (MW 193.11 gmol^−1^). MS for peak 1 at ca. 5.52 min showed a molecular ion at m/a 58 and an ion fragment at *m*/*z* 136, both of which are commonly published as a precursor fragment of MDMA under GCMS analysis. The lack of resolution may indicate signs of co-elution of two or more possible compounds, or that this was a particularly highly adulterated sample. These findings were further confirmed by UPLC-MS.

The NMR analysis resulted in a clear spectrum with defined peaks within the aromatic, splitting, and aliphatic regions, although some overlapping was suspected within the splitting and aliphatic regions, specifically ca. 6.78, 5.91, and 2.68 ppm. Peaks detected between ca. 6.8–7.5 ppm were indicative of aromatic structures, but the frequency and number of signals present in the spectra were not indicative of any single compound. Compared to the literature, signals at ca. 6.70, 5.91, 2.6–3.4, and 1.2 ppm were indeed indicative of the MDMA molecule in the literature [59] and could be used to integrate the NMR for this compound; therefore, some comparisons can be made. A single proton showing at ca. 3.3 ppm was clear and indicative of the CH on the aliphatic chain nearest to the NH, although we would expect to see two additional singlet methyl-proton peaks at either end of the molecule, which were not visible.

Sample S8 was presented as an LSD spaceship (Figure 2). The combined analytical techniques reported inconclusive results for this sample.

Sample S4 was presented as heroin powder. The client reported it was too weak to produce an effect as compared with previous batches. The presented powder was coarsely granular and dark yellow in appearance. The Raman response indicted a suspected taurine content (CC > 66%). The GC-MS analysis showed a dominant peak at 13.3 min reporting a nominal mass of *m*/*z* 327, resulting in a NIST library match of 60.3% for 6-monoacetyl morphine (6-MAM), one of the three active metabolites of heroin. The tested compound also showed an array of additional peaks, and analysis of their associated fragmentation patterns suggested the sample contained multiple compounds, as expected with street-purchased heroin. 6-MAM often presents either as base (MW 369.4 gmol^−1^) or as hydrochloride (MW 405.9 gmol^−1^), although GC-MS analysis was unable to identify either of these parent-ion masses; however, documented reference-standard data showed a high fragmentation-pattern match when compared to the dominant peak at 13.3 min, in support of the presence of 6-MAM in this sample. The UPLC-MS analysis also confirmed the presence of 6-MAM. The analysis detected a region of multiple chromophore peaks, between 0.39 and 0.60 min, where four distinct signals were visible, with the loss of some peak resolution between 0.39 and 0.40 min, along with additional peaks at 2.55 min and 3.56 min. The MS for each peak was investigated and found to offer differing responses in terms of both nominal mass *m*/*z* and λmax (Table 2). The presence of multiple peaks with differing responses indicates this compound to be a highly complex mixture of compounds.

The NMR analysis was inconclusive due the complexity of the sample and possibly the low content of the active drug. The presented sample showed an array of proton signals in both the aromatic and aliphatic regions, as would be expected from an illicit compound; however, due to the complexity of the spectra and the abundance of overlapping of signals, specifically across the aliphatic region, it was not possible at this stage to assign any functional groups with any certainty. Doublet and multiplet peaks throughout the region ca. 7.15–7.8 ppm seemed characteristic of aromatic protons within both the base and hydrochloride variants of heroin. However, without the ability to assign the functional groups across the aliphatic region, determining each structure was not possible.

To our knowledge, this was the first study that evaluated the use of handheld Raman spectroscopy in a Home Office-licensed pharmacist-led drug-checking service within a community SMS.

The Raman analysis served as a screening tool that accurately reported the sample content in relatively pure samples, as demonstrated by the cocaine samples. The Raman analysis of formulated products such as tablets containing high amounts of traditional excipients such as lactose and microcrystalline cellulose failed to detect the active ingredient, which may have been present in small amounts. This was demonstrated by the analysis of the benzodiazepine tablets. The three wet analytical techniques were superior to Raman spectroscopy in their performance but still failed to detect the content of four out of 13 compounds, and two out of three wet analytical techniques (i.e., UPLC-MS and NMR) failed to detect three out of 13 compounds. Limitations related to the wet analytical techniques were mainly due to poor sample concentration (higher drug concentrations are particularly required for poor-sensitive NMR instruments), the presence of previously unknown compounds, and the presence of complex mixtures.

The use of handheld Raman spectroscopy meant that the on-site service delivery was truly rapid and portable: the equipment was easy to store securely and took up minimal space. Additionally, the functional setup was easy to achieve in a timely manner within an existing busy SMS environment without the need for building works: this is important when considering operational service delivery, especially when there may be limited space and funding available and where the rooms may otherwise be needed for “normal” SMS-care delivery. The ability for Raman spectroscopy to provide results at a level that was sufficient for checking in a timely manner on small traces of samples was also important in this setting, where individuals may be unwilling to provide large sample volumes, and they did not have to wait long to receive their results.

Cocaine was presented for analysis multiple times, indicating its relative prevalence and given that interventions for cocaine use do not always require formal interventions, highlighting the value of drug checking as an engagement tool for people who may not otherwise access SMS [60]. Additionally, this accentuates the need for increased use of drug checking embedded within SMS to enable more timely identification of new trends and any associated drug alerts.

A significant limitation of this study was the limitation of the sample size, and further studies are required to confirm our findings; however, we were able to demonstrate proof of concept. The limitations of Raman spectroscopy were mainly associated with the detection of the psychoactive substance(s) in complex mixtures and have been similarly identified by others [39,52].

## 4. Conclusions

Whilst acknowledging our small sample size, handheld Raman spectroscopy is a relatively effective method for drug checking in the context of a harm-reduction intervention provided in community SMS. Other analytical techniques, for example, UHPLC-MS, GC-MS, and ^1^H NMR, may be preferable for confirmatory analyses; however, these present with logistical challenges in terms of analysis in the field, and, due to poor sensitivities in some instruments and limits of detection (LOD) limitations that accompany wet analytical techniques, they may require samples of a larger mass to be surrendered for combined analyses; which could offer added complexities within a clinical setting. The challenges of accurately identifying substances from complex mixtures can be experienced across all analytical techniques: ideally, more than one technique should be used to triangulate findings. Future work should focus on trialling ion-mobility spectrophotometers for non-destructive drug detection in community settings. Home Office-licensed drug-checking services using such approaches should be rolled out further and embedded into community SMS delivery to improve SMS engagement, timeliness, and availability of drug alerts and help reduce associated potential drug-related harm.

## 5. Limitations

The authors do recognise the limitations of this study due to the small sample size. It is important to highlight that client recruitment in this study was limited due to multiple reasons, including local political elections, client concerns of being arrested if found to be in possession of possible Schedule 1 controlled substances, and client concerns that analysis results could have been added to their records, which may have had an impact on them if they were clients referred through the criminal-justice route.

Another limitation to that study is the absence of a control group. This is because drug checking is not common practice, and this pilot was the first of its kind to be conducted within a substance-misuse service. Screening for drugs within these services is usually undertaken through urine analysis using presumptive tests that screen for a limited set of drugs, e.g., prescribed medications such as methadone and buprenorphine, as well as recreational drugs such as cocaine, heroin, amphetamine, benzodiazepine, and cannabis. These tests do not routinely screen for novel psychoactive substances.

## Figures and Tables

**Table 1:** Summary of received drug samples.

Sample ID	Sample Type	Original Sample Mass (g)	Mass of Sample Tested (g)
S1	Herb	0.0526	0.0526
S2	Tablet	0.2093	0.0101
S3	Powder traces	*	*
S4	Powder	0.0124	0.0124
S5	Tablet	0.1451	0.1451
S6	Powder	0.0023	0.0023
S7	Tablet	0.2342	0.2342
S8	Paper	0.0033	0.0033
S9	Powder	0.2100	0.2100
S10	Powder	0.5089 **	0.5089 **
S11	Tablet	0.1790	0.1790
S12	Tablet	0.2857	0.2857
S13	Half a tablet	0.2106	0.2106

* Unable to record due to retrieved sample size; ** including surrendered baggy due to sample type.

**Table 2:** Summary of qualitative and analysis of surrendered samples using handheld Raman spectroscopy, GC-MS, UHPLC-MS, and 1^H^ NMR.

	Handheld Raman Analysis (λ_ex_ = 1064 nm)		GCMS				UHPLC				1H-NMR (600 MHz, METHANOL-D3)	
Amnesty Declaration (Sample ID)	Raman ID	Valid Hit (Rigaku Mixtures Algorithm CC Value)	Retention Time (min)	Molecular Ion m/z	Ion-Fragmentation Pattern Confirmed in Literature	NIST ID	Retention Time (min)	Base Peak m/z	Ion-Fragmentation Pattern Confirmed in Literature	Confirmed ID (Using Ref Std, SWGDRUG Database, and/or Literature)	J-Coupling Report (Couplings Matched Using Ref. Standard and/or Literature)	Confirmed ID (Ref. Standard or Literature)
Stimulants												
Cocaine (S3)	Cocaine Hydrochloride	0.98	11.041	82	272, 198. 182, 151, 94, 82	Cocaine	0.53	304.5	182.5, 326.4, 229.5, 117.2	Cocaine	*7.94–7.93* (*m*, *2H*), *7.64–7.61* (*m*, *1H*), *7.50–7.47* (*m*, *2H*), *5.56* (*d*, *J* = *9.6 Hz*, *1H*), *4.18* (*s*, *1H*), *3.98* (*s*, *1H*), *3.64* (*d*, *J* = *7.6 Hz*, *3H*), *3.56* (*s*, *1H*), *3.33* (*d*, *J* = *6.2 Hz*, *1H*), *2.84* (*s*, *2H*), *2.49–2.38* (*m*, *4H*), *2.19* (*t*, *J* = *10.0 Hz*, *2H*)	Cocaine
Cocaine (S6)	Cocaine Hydrochloride	0.95	11.024	303	272, 198. 182, 151, 94, 82	Cocaine	0.53	304.5	182.5, 326.4, 229.5, 117.2	Cocaine	*δ 7.94* (*dd*, *J* = *8.1*, *1.5 Hz*, *2H*), *7.70* (*d*, *J* = *8.2 Hz*, *0H*), *7.63* (*t*, *J* = *7.2 Hz*, *1H*), *7.50–7.47* (*m*, *2H*), *5.56* (*d*, *J* = *9.6 Hz*, *1H*), *4.25–4.17* (*m*, *1H*), *3.98* (*s*, *1H*), *3.66–3.62* (*m*, *3H*), *2.84* (*s*, *3H*), *2.50–2.38* (*m*, *4H*), *2.19* (*s*, *2H*)	Cocaine
Cocaine (S9)	Cocaine Hydrochloride	0.97	11.021	304	272, 198. 182, 151, 94, 82, 78	Cocaine	0.53	304.5	182.5, 326.4, 229.5, 117.2	Cocaine	*δ 7.94–7.93* (*m*, *2H*), *7.64–7.61* (*m*, *1H*), *7.50–7.47* (*m*, *2H*), *5.57* (*d*, *J* = *10.3 Hz*, *1H*), *4.62* (*s*, *0H*), *4.18* (*s*, *1H*), *3.98* (*s*, *1H*), *3.64* (*s*, *3H*), *3.57* (*d*, *J* = *5.5 Hz*, *1H*), *2.85* (*s*, *3H*), *2.49–2.38* (*m*, *4H*), *2.20* (*t*, *J* = *10.7 Hz*, *2H*)	Cocaine
Stimulant (S10)	Caffeine Creatine	0.82	8.22	194	195, 165, 122, 94, 82, 67, 56	Caffeine	0.57	195.4	195.4, 174.2, 149.1, 121.4, 117.5, 101.7, 100.5	Caffeine	*δ 7.85* (*s*, *1H*), *3.95* (*s*, *3H*), *3.51* (*s*, *3H*), *3.33* (*d*, *J* = *2.7 Hz*, *3H*)	Caffeine
SCRAs												
Spice (S1)	NC	0.76	13.461 15.108	220 376	NCNC	7.53% Cyano (decahydroisoquinolin-3-ylidene)-acetic acid, t-butyl ester C16H24N2O2 60.0% Isophthalic acid, hexyl 1-naphthyl ester C24H24O4	3.69	232.5 (775.4 Including adducts)	NC	NC	NC	NC
Depressants												
Benzodiazepine (S2)	Starch Talc Carboxy-methylcellulose	0.69	16.579	343	344, 343, 315	Etizolam	2.36	343.3	346.3, 345.4, 343.3	Etizolam	*δ 7.51–7.41* (*m*, *4H*), *6.46* (*s*, *1H*), *4.62* (*s*, *1H*), *2.85–2.81* (*m*, *2H*), *2.69* (*s*, *3H*), *1.57* (*t*, *J* = *7.2 Hz*, *4H*)	Etizolam
Valium (S7)	Talc 5-Iodo-2-aminoindane Diazepam	0.63	16.55	343	343, 344, 316	Etizolam	2.6	343.5	316, 313, 224, 208, 137, 75	Etizolam	*δ 7.50–7.41* (*m*, *4H*), *6.46* (*d*, *J* = *1.4 Hz*, *1H*), *2.85–2.81* (*m*, *2H*), *2.69* (*s*, *3H*), *1.56* (*q*, *J* = *7.1 Hz*, *4H*)	Etizolam
Valium (S5)	Lactose Amphetamine analogues	0.63	12.66	275	304, 275, 241, 150, 112, 75	Delorazepam	2.4	321.3	321.3, 323.2, 343.3, 345.3, 303.3, 229.3, 150.0, 101.5,	NC	NC	NC
Valium (S11)	α-Lactose monohydrate	0.88	12.84	284	256, 260, 221, 177, 165, 151, 110, 77, 51	Diazepam	2.84	285.4	285, 257, 256, 241, 228, 221, 207, 179, 150, 117	Diazepam	*δ 7.64* (*dd*, *J* = *8.6*, *2.4 Hz*, *1H*), *7.50–7.56* (*m*, *4H)*, *7.43–7.46* (*m*, *2H*), *7.21* (*s*, *1H*), *3.39* (*d*, *J* = *7.6 Hz*, *3H*)	Diazepam
Xanax bar (S12)	Microcrystalline cellulose	0.77	11.03 11.70 12.30	92 213 287	NC NC	Acetaldehyde, phenylhydrazone C8H10N2 Promethazine C17H20N2S Benzenemethanol, 4-amino-α,α-bis (4-aminophenyl)-C19H19N3O	0.45 0.63 0.73 1.86	229.2 286.6 229.4 288.4	NC	NC	NC	NC
Other												
Unknown (S13)	No response	N/A	5.52 5.63	58 149	136, 77, 58	N-Methyl-3,4-methylenedioxyamphetamine	0.44	194.4	103, 105, 121, 135, 194	N-Methyl-3,4-methylenedioxyamphetamine	NC	NC
LSD (S8)	No response	N/A	NC	NC	NC	NC	0.62	324.4	NC	NC	NC	NC
Heroin (S4)	Taurine	0.66	13.3	327	328, 285, 216, 147, 94	6-MAM	0.49	328.3	328.1, 165.05	6-MAM	NC	NC

Abbreviations: N/A (not applicable); NC (Not Confirmed); 6-MAM (6-monoacetylmorphine).

## Data Availability

Data supporting reported results can be found with the corresponding author.

## Supplementary Materials

The following supporting information can be downloaded at: https://www.mdpi.com/article/10.3390/ijerph20064793/s1, Raman peak assignments related to Figure 2 were confirmed from published literature and compiled as Supplementary Materials.
