# Supplementary material for: Handheld Raman Spectroscopy in the First UK Home Office Licensed Pharmacist-Led Community Drug Checking Service
Source: Int J Environ Res Public Health. 2023 Mar 8;20(6):4793. doi: 10.3390/ijerph20064793 (PMC10049009; doi:10.3390/ijerph20064793)
Supplement: Supplementary file 1 [file ijerph-20-04793-s001.zip › ijerph-2186436-supplementary.pdf]

Supplementary Materials:

Table S1. Raman peak assignments for both cocaine and diazepam

Literature data from Penido et. Al. 2016 [59]

COCAINE				
**Functional groups**	**Mode**	**Literature peaks (cm^{-1})**	**Rigaku peaks (cm^{-1})**	**Renishaw peaks (cm^{-1})**
C–C tropane ring	Stretching	~898	~899 w	~870 m
C–C tropane ring	Stretching	~874	~870 m	~896 w
Aromatic ring	Symmetric stretching –breathing	~1004	~1000 s	~1000 s
Aromatic ring	Asymmetric stretching	~1026	~1028 m	~1026 m
C–N	Stretching	~1279	~1274 m/w	~1278 m/w
CH3	Asymmetric deformation	~1462	~1460 w	~1459 w
C=C	Ring stretching	~1601	~1596 vs	~1598 s
C=O	Symmetric stretching	~1716	~1716 s	~1716 s

Literature data from Gunaserakan et al., 2006 [60]

DIAZEPAM				
**Functional groups**	**Mode**	**Literature peaks (cm^{-1})**	**Rigaku peaks (cm^{-1})**	**Renishaw peaks (cm^{-1})**
C–N–C	Asymmetric bending	~698 vw	~695 m/w	~690 m
_	_	_	~1000 m	~998 w/m
C–C	Stretching	~1321 vw	~1315 s	~1312 s
C–N	Symmetric stretching	~1174 vw	~1169 m	~1168 m
C-C aromatic ring	Asymmetric stretching	~1561 m/w	~1567 w	~1562 w
C=N	Stretching	~1599 vs	~1594 vs	~1593 vs
C=O	Stretching	~1690 w	~1689 m/w	~1685 m/w
